# Engineering a Multi-Dimensional Thermally Conductive Network to Enhance the Thermo-Mechanical Stability of Concrete

**DOI:** 10.3390/ma19194203

**Published:** 2026-10-01

**Authors:** Chen Chen, Xin Liu, Hui Xie, Lijing Shao, Long Yuan, Pan Feng

**Affiliations:** 1 School of Materials Science and Engineering, Southeast University, Nanjing 211189, China; ccchen@seu.edu.cn (C.C.); xieh@seu.edu.cn (H.X.); 230228659@seu.edu.cn (L.S.); 230258364@seu.edu.cn (L.Y.); 2 State Key Laboratory of Engineering Materials for Major Infrastructure, Nanjing 210008, China

**Keywords:** thermal conductivity, steel fibers, graphite-modified recycled aggregates, thermo-mechanical properties, thermal treatment

## Abstract

Reliable thermo-mechanical performance is typically required in intensive thermal management applications, such as thermal energy storage systems and concrete-structured data centers; however, there is usually a trade-off between thermal conductivity and strength when designing thermally conductive concrete. To address the critical demand for concrete that combines high thermal conductivity with excellent structural stability after thermal loading, a novel multi-dimensional thermally conductive network was constructed by incorporating one-dimensional steel fibers and two-dimensional graphite-modified recycled aggregates to form a synergistic composite (SFRHCC). Results demonstrate that the thermal and mechanical stability of SFRHCC subjected to 200 °C was substantially improved compared with the unmodified reference. Specifically, after thermal treatment at 200 °C, SFRHCC restricted its thermal conductivity loss to only 7.6%, in stark contrast to the 28.2% reduction observed in ordinary recycled aggregate concrete (RAC). Furthermore, SFRHCC retained 92.0% and 91.3% of its reference compressive and splitting tensile strengths, vastly outperforming the unmodified reference (which retained only 75.0% and 72.4%, respectively). Both X-ray computed tomography (X-CT) and Euclidean distance transform (EDT) characterizations elucidate a high-density linear-planar overlapping network with an overall spatial connectivity of 9.94%, where the soft graphite coating layer accommodates thermal mismatch deformations and restricts volumetric fluctuations. This structural integrity effectively transforms the interfacial failure mode from simple weak-interface debonding to cohesive matrix tearing. These findings offer a promising materials design strategy for developing structural-functional integrated concrete for thermal management applications.

## 1. Introduction

As the cheapest and most widely used building material in the world, concrete is often employed in various facilities and complex application environments. With the rapid development of technologies involving intensive thermal management, such as high-temperature thermal energy storage systems and concrete-structured data centers [1], there is an increasing demand for concrete that combines high thermal conductivity with excellent mechanical stability during and after thermal exposure or thermal shocks [2]. Conventional concrete, however, generally exhibits relatively low thermal conductivity and pronounced temperature-induced deterioration, which can hinder efficient heat dissipation and compromise structural integrity under severe thermal conditions [3,4,5]. Therefore, developing high-performance concrete capable of simultaneously facilitating efficient heat transfer and maintaining structural stability under complex thermal environments has become increasingly important.

To enhance the thermal conductivity of concrete, various highly thermally conductive constituents have been incorporated to construct interconnected heat-transfer pathways, including steel fibers [6,7], graphite [8,9], thermally conductive aggregates [10], and carbon nanotubes [11,12], among others. For instance, Lee and Kwon [10] pointed out that when magnetite is used to partially replace conventional aggregates, the enhancement in thermal conductivity is extremely limited if the volume replacement ratio is below 30%. However, increasing the volume replacement ratios of coarse and fine aggregates to 90% and 50%, respectively, can result in a 2.3-fold increase in thermal conductivity. Nevertheless, the incorporation of thermally conductive fillers does not necessarily lead to a simultaneous improvement in mechanical performance; in many cases, the introduction of conductive phases can adversely affect the mechanical properties of concrete due to increased interfacial heterogeneity, poor dispersion, or the formation of weak interfaces [13]. More importantly, most existing studies of high thermal conductivity concrete have primarily focused on thermal conductivity, while comparatively limited attention has been paid to its structural stability during and after intensive heat transfer or thermal exposure. It is essential for high thermal conductivity concrete to maintain adequate mechanical integrity after exposure to thermal loads. Therefore, evaluating the residual mechanical properties and thermal transport stability of high thermal conductivity concrete after thermal exposure is essential for assessing its reliability and practical applicability in complex thermal environments.

When concrete is exposed to elevated temperatures, a series of physicochemical and thermo-mechanical changes occur, leading to progressive degradation of its thermal and mechanical properties. The evaporation of free water and the dehydration or decomposition of hydration products, such as ettringite (AFt) and calcium silicate hydrate (C-S-H), induce pore pressure development and microcracking within the cementitious matrix [14,15,16]. Meanwhile, the mismatch in thermal expansion between the cement paste and aggregates can generate substantial internal stresses and promote crack initiation and propagation [17]. The temperature gradient between the exposed surface and the concrete interior further intensifies these thermal stresses, potentially resulting in severe cracking and even surface spalling at elevated temperatures [18]. These damage processes induce localized stress concentrations and progressively deteriorate the macroscopic mechanical integrity of concrete. From another perspective, the incorporation of thermally conductive phases may provide additional mechanisms for mitigating such thermal damage. Steel fibers can physically bridge and restrain the propagation of macrocracks [19], while graphite, owing to its layered structure and relatively low thermal expansion, may accommodate thermally induced deformation through interlayer sliding [20]. Therefore, elucidating how different thermally conductive fillers influence the thermal damage evolution and residual mechanical performance of concrete is of great significance. Such understanding can provide fundamental guidance for the design of high thermal conductivity concrete with enhanced thermal stability and crack resistance for applications involving thermal loading.

This study investigates the thermo-mechanical properties of concrete incorporating different thermally conductive fillers after thermal treatment ranging from 25 to 200 °C. This specific range targets the typical service conditions of moderate thermal environments, such as data centers and thermal energy storage. The selected gradient (80, 150, and 200 °C) systematically corresponds to progressive thermal damage phases—from free water mobilization and rapid evaporation to severe thermal mismatch—prior to the massive chemical decomposition of cement hydration products. The thermal stability and damage of the composites were evaluated based on the residual thermal conductivity, compressive strength, and splitting tensile strength. Furthermore, the enhancement mechanisms of the multi-dimensional thermally conductive network are elucidated through a combination of approaches. These include the characterization of continuous thermal expansion deformation during heating, the application of X-ray computed tomography (X-CT) [21] combined with Euclidean distance transform (EDT) to reconstruct and quantitatively evaluate the three-dimensional connectivity of the thermally conductive network, and the use of SEM-EDS to interrogate the interfacial contact morphology and micro-damage characteristics. Through these analyses, this study systematically reveals the distinct roles of graphite and steel fibers in regulating thermal conduction, thermal deformation, microcrack suppression, and mechanical retention in concrete. The findings provide new insights into the thermo-mechanical enhancement of concrete and establish a materials-design strategy for developing high-performance concrete capable of efficient heat transfer while maintaining structural integrity under thermal loading.

## 2. Materials and Methods

### 2.1. Materials

The cementitious matrix was prepared using a commercial P·I 42.5 ordinary Portland cement and tap water, with standard natural river sand selected as the fine aggregate. For the coarse aggregate, recycled coarse aggregates (RCAs) produced by the crushing and sieving of C30 concrete were sourced from Beijing Urban Green Source Environmental Protection Technology Co., Ltd. (Beijing, China). The RCAs exhibit a continuous gradation with a particle size ranging from 5 mm to 16 mm. Their apparent density, water absorption rate, crushing value, needle and flake particle content, and old mortar content are 2660 kg/m^3^, 6%, 12%, 5%, and 0.8%, respectively. To construct the multi-dimensional thermally conductive network, two types of functional fillers were employed. Multilayer lamellar graphite, provided by Henan LG Graphite Co., Ltd. (Zhengzhou, China), was utilized for aggregate coating. It has a particle size of 150 μm, a high thermal conductivity of 141.1 W/(m·K), and a purity of ≥99.0% with trace amounts of Al. Additionally, straight steel fibers with a length of 5 mm, a diameter of 0.2 mm, and a thermal conductivity of approximately 50 W/(m·K) were supplied by Panjin Hengshui Building Materials Co., Ltd. (Hengshui, China).

### 2.2. Mixture Design and Specimen Preparation

To systematically investigate the synergistic mechanism of multiphase components, four concrete mixture systems were designed, with their proportions detailed in Table 1. The basic ordinary recycled aggregate concrete is denoted as RAC. To evaluate the modification effect of single rigid materials, straight steel fibers with a volume fraction of 3.5% were added to the RAC to prepare SFRAC. An L/D ratio of 25 and a fiber dosage of 3.5 vol% were adopted based on our previous systematic optimization study [22], which demonstrated that this configuration achieves an optimal balance between constructing an efficient thermally conductive network and maintaining adequate macroscopic mechanical properties. To assess the effect of the graphite coating layer, graphite-modified recycled aggregate concrete is denoted as GMRAC. The graphite-modified recycled aggregates were prepared following the RA11B method detailed in the study by Chen et al. [8]. Specifically, a one-pot coating method was employed. First, the graphite and cement (at a mass ratio of 1:1) were homogenously mixed in a blending machine to produce the dry coating materials. Subsequently, the unmodified recycled coarse aggregates, the coating materials, and half of the total mixing water were added to a coating machine and stirred for 1 min to achieve initial wetting. The remaining half of the water was then introduced and stirred for an additional 3 min to ensure a uniform coating layer. Finally, the modified aggregates were cured for 7 days under standard conditions (20 ± 2 °C, 64 ± 5% relative humidity) and sieved through a 2.36 mm mesh to remove uncoated residues. Finally, the synergistically modified composite, incorporating both the graphite coating layer and steel fibers, is denoted as SFRHCC.

**Figure 1:**
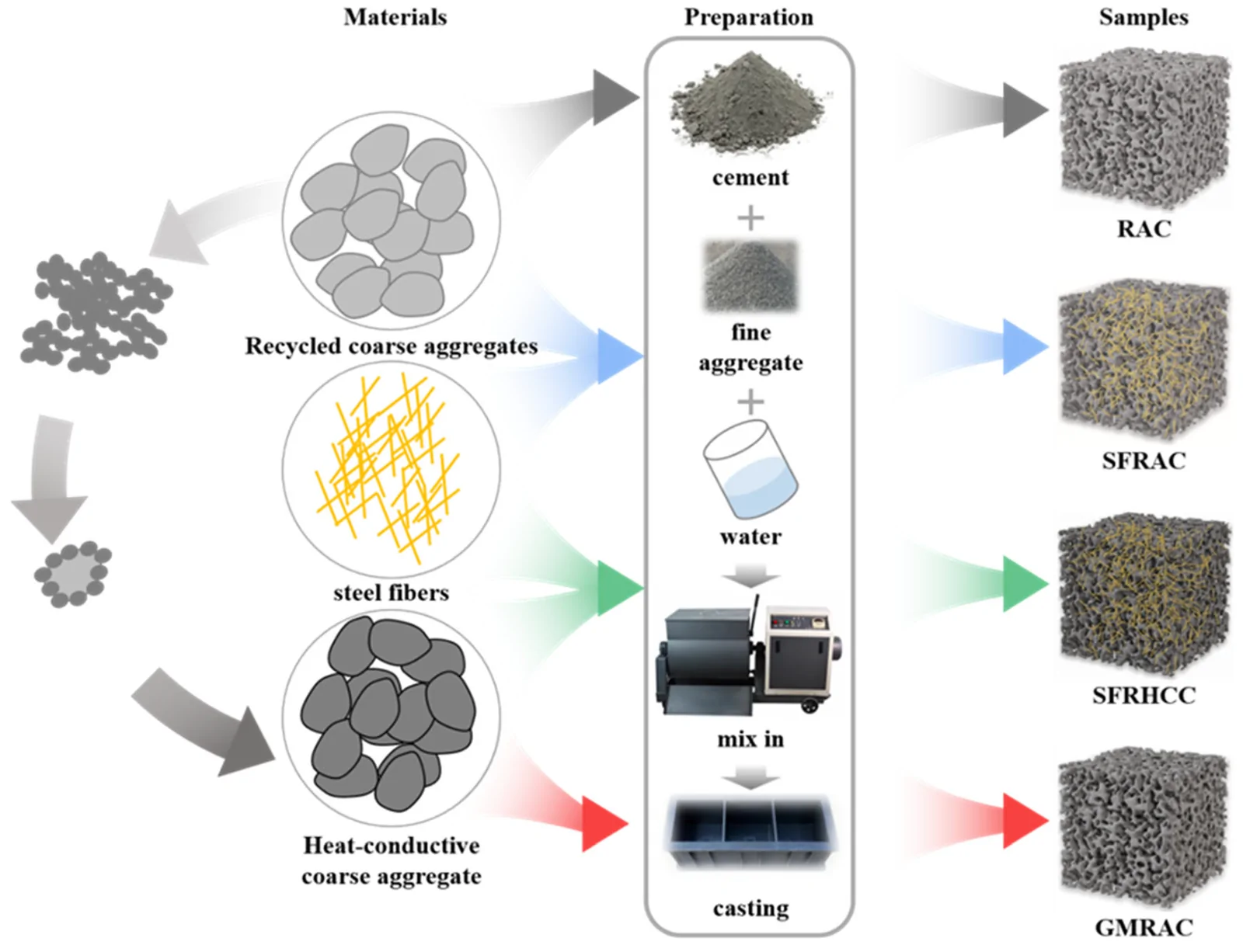
Schematic of the preparation and casting process for various concrete systems.

All four groups utilized the same amount of cement, water, and fine aggregates, maintaining strict variable control over the coarse aggregate surface state and the addition of steel fibers. Specifically, all recycled coarse aggregates were prepared in a pre-saturated surface-dry condition prior to mixing to ensure a constant effective water-to-cement ratio. A schematic overview of the preparation and casting process for the various concrete systems is presented in Figure 1. Since the water-to-cement (w/c) ratio was strictly fixed at 0.60 across all groups without adjustments for flowability variations (to preserve the intrinsic porosity and strength of the baseline cementitious matrix), the fresh mixture was cast into 100 × 100 × 100 mm^3^ plastic molds and subjected to mechanical vibration for 1 min. This extended vibration was specifically applied to overcome the flowability reduction induced by the high fiber content and ensure robust compaction. The samples were demolded after 24 h, followed by standard curing at 20 °C ± 2 °C and a relative humidity of over 95% until the testing age.

### 2.3. Characterizations

#### 2.3.1. Thermal Conductivity

The thermal conductivity of the concrete was measured using a DRE-III multi-functional rapid thermal conductivity tester based on the transient plane source (TPS) method. The equipped Hot Disk probe possesses a physical diameter of 15 mm, yielding a measurement zone-to-maximum aggregate size (MAS = 16 mm) ratio of approximately 0.94. Prior to testing, the specimens were removed from the curing room and dried to a constant weight at 60 °C. This strictly oven-dried state was adopted to eliminate the variable influence of free water within the pores, thereby ensuring that the measured data accurately reflects the intrinsic heat-transfer capability of the solid-phase multi-dimensional network under the targeted high-temperature conditions. During the measurement, the probe was sandwiched between two identical specimens. To minimize the interfacial thermal contact resistance caused by the air layer, the contact surfaces of the specimens were polished smooth beforehand to ensure an intimate contact between the probe and the material. Furthermore, to eliminate the potential influence of localized heterogeneity within the composite system on the test results, the contact position of the probe on the specimen surface was altered after each individual measurement. Three independent tests were conducted for each sample group, and the average value was adopted as the representative thermal conductivity. All measurements were performed at a constant temperature of 25 °C.

#### 2.3.2. Thermal Expansion Behavior

To evaluate the dimensional stability and deformation characteristics of the concrete during the heating process, the thermal expansion behavior of each group was measured using a TMA 403 F3 thermo-mechanical analyzer. The test samples were cut from the specimens cured to the designated age, with dimensions not exceeding 25 mm in height and 10 mm × 10 mm in cross-section. To guarantee the accuracy of the displacement measurements, both the top and bottom surfaces of the samples were polished flat and strictly parallel to each other. The temperature program was set to increase from room temperature (25 °C) to 200 °C at a constant heating rate of 1 °C/min. During the heating process, the longitudinal expansion displacement of the samples was continuously recorded. For each mixture system, three independent specimens were tested to ensure reproducibility. In the data processing stage, the recorded absolute displacements were converted into relative expansion strains. By calculating the mean values and standard deviations of the three replicate samples at each temperature node, thermal expansion strain–temperature curves with error bands were plotted to objectively reflect the overall thermal expansion trends and the data dispersion of the materials.

#### 2.3.3. Mechanical Properties

The compressive and splitting tensile strengths of the concrete were evaluated using a TYE-2000B universal testing machine, strictly following the guidelines of the GB/T 50081-2019 standard [23]. Based on the mixture proportions outlined in Table 1, cubic specimens with dimensions of 100 × 100 × 100 mm^3^ were cast and subjected to a standard 28-day curing period. Prior to thermal exposure, the cured specimens were dried to a constant weight at 60 °C to establish a uniform moisture baseline. Subsequently, the specimens were placed in an electric oven at room temperature and heated gradually to the target temperatures (80 °C, 150 °C, and 200 °C) to mitigate initial thermal shock. After maintaining the target temperature for 3 h, the oven was turned off, and the specimens were allowed to cool naturally to ambient temperature within the closed oven. This slow cooling procedure was strictly adopted to preclude secondary structural cracking induced by sudden thermal gradients. Following the designated thermal treatments, the specimens were evenly distributed to undergo different mechanical assessments: three replicates were designated for the compressive strength test, and the remaining three were utilized for the splitting tensile test at each target temperature. During the execution of the measurements, constant loading rates of 5 kN/s and 0.5 kN/s were applied for the compression and splitting tension tests, respectively. To ensure the reliability of the experimental data, the ultimate strength values reported for each specific mixture configuration represent the arithmetic mean of the three corresponding replicates.

#### 2.3.4. X-Ray Computed Tomography (X-Ct) and 3d Image Analysis

To precisely acquire the 3D spatial distribution characteristics and comprehensively assess the connectivity of the highly heat-conductive phases both qualitatively and quantitatively, non-destructive testing was performed using X-ray computed tomography (X-CT). Based on the proportions detailed in Table 1, four sets of cylindrical concrete samples (φ50 mm × 100 mm) were prepared. After a standard 28-day curing period, the internal microstructures were examined using a Y.CT PRECISION S X-CT scanner(YXLON International, Hamburg, Germany). The scanning was conducted in cone-beam mode with a working voltage range of 10 to 225 kV.

The acquired raw data were imported into Dragonfly software (Version 2022.2) for 3D image reconstruction and processing. To ensure consistency and accuracy across different specimens, the reconstructed images were standardized to a grayscale range from 0 (black) to 255 (white) with an image matrix size of 1024 × 1024 pixels. The X-CT scanning achieved a voxel spatial resolution of approximately 50 μm, which is sufficient to capture the macroscopic spatial distribution of the steel fibers and the coated aggregates. The phase segmentation procedure relied on grayscale thresholding driven by the distinct density contrasts among the constituents: the high-density steel fibers exhibited the highest brightness, followed by the cementitious matrix, while the air voids appeared as the darkest regions. Furthermore, inherent methodological uncertainties during segmentation, such as the partial volume effect at phase boundaries, were strictly mitigated by calibrating the grayscale thresholds against the known volumetric fractions of the raw materials. Based on this rigorously calibrated segmentation, the spatial volumes of the target phases were accurately extracted, thereby obtaining the regions of interest for the steel fibers (ROI-fiber) and the graphite coating layer (ROI-graphite).

Following the precise extraction of the target phases, the data were exported to Avizo software (Version 2021.1). The built-in Axis Connectivity module was utilized to isolate and extract the continuous, physically overlapping thermally conductive networks from the discrete, isolated phases. By automatically identifying contacting voxels in 3D space, this module enables the quantitative calculation of the connected phase volumes using Equation (1) [24,25]:

(1) $$C=\frac{G}{V}\times 100\%$$

where *C* represents the spatial connectivity rate of the thermally conductive phases (%); *G* denotes the volume of the thermally conductive phases that overlap to form a continuous network within the matrix (m^3^); and *V* is the total volume of the thermally conductive phases within the analyzed unit (m^3^).

Furthermore, to quantitatively characterize the short-range contact features between the different thermally conductive fillers in 3D space, the EDT method was introduced to compute the minimum spatial distance from the surface of the steel fibers to the adjacent graphite coating layer. Based on the segmented ROIs, the Surface Determination function in Dragonfly was employed for 3D model reconstruction. The previously extracted ROIs were utilized as masks to define the starting points for the surface search, generating high-precision surface mesh models denoted as Mesh-fiber and Mesh-graphite. The algorithm then calculated the shortest straight-line distance from each vertex on the Mesh-fiber to the surface of the Mesh-graphite. Ultimately, these shortest distance values were mapped onto the entire surface of the Mesh-fiber, generating a global spatial distance distribution rendered with pseudo-color 3D visualization. This EDT methodology provides a precise geometric characterization for evaluating the synergistic overlapping status of the multi-dimensional fillers.

#### 2.3.5. Microstructural Morphology and Elemental Analysis

The microstructural morphologies and elemental compositions of the concrete samples were characterized using a Nova NanoSEM 450 field-emission scanning electron microscope (SEM) equipped with an energy-dispersive X-ray spectrometer (EDX). After a standard 28-day curing period, representative fragments were obtained by crushing the concrete specimens. To arrest the cement hydration process via solvent exchange, these fragments were immediately immersed in isopropanol for 2 days. Subsequently, the samples were vacuum-dried at 40 °C until a constant weight was achieved. Prior to microscopic observation, a thin layer of platinum was sputtered onto the sample surfaces for 90 s using a Q150T S high-resolution sputter coater. This conductive coating was applied to effectively prevent surface charging and ensure high-quality imaging during the SEM analysis.

## 3. Results and Discussion

### 3.1. Residual Compressive Strength After Thermal Treatment

**Figure 2:**
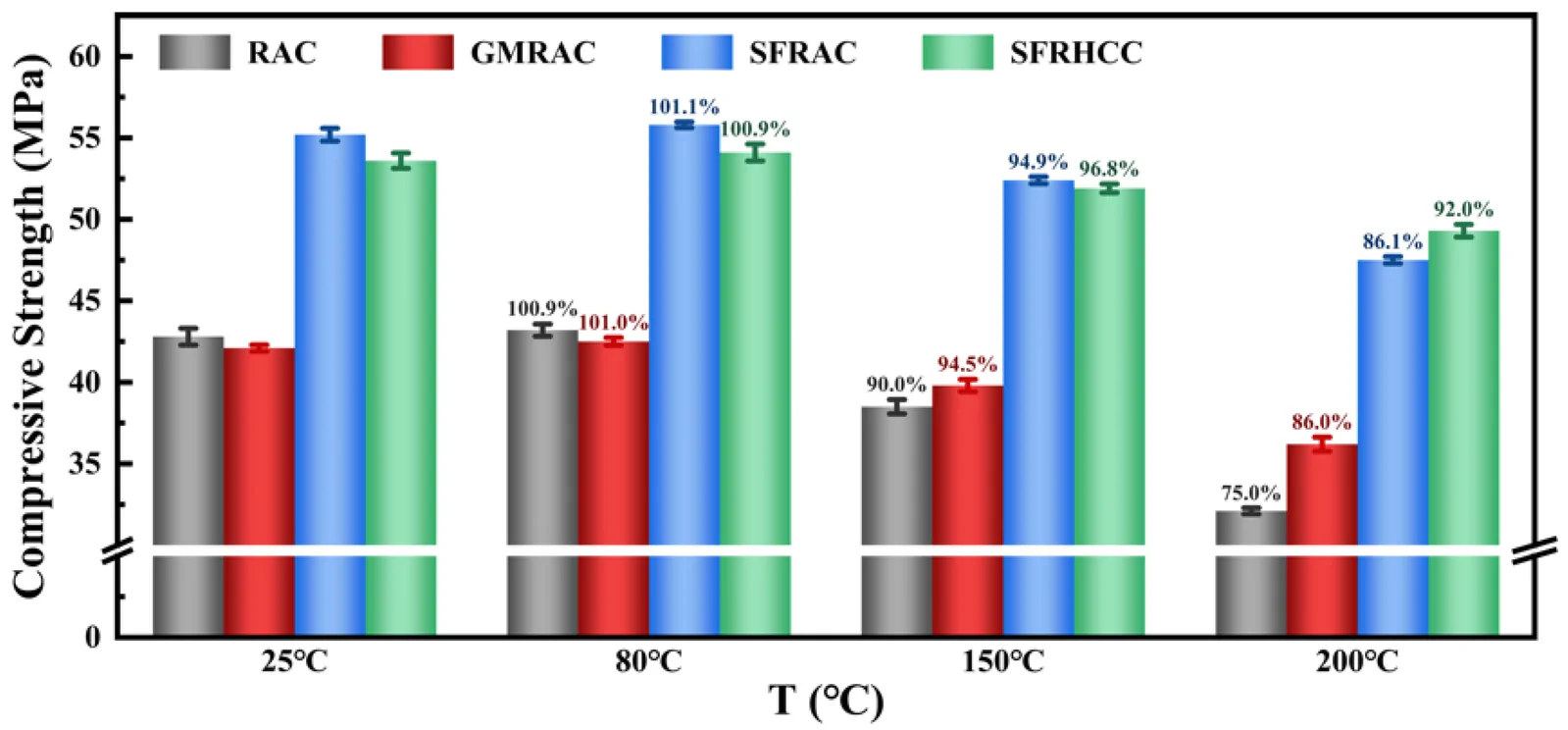
Compressive strength and strength retention rate of various concrete systems at 25, 80, 150, and 200 °C.

Residual compressive strength is a key indicator for evaluating the structural safety and mechanical stability of cement-based composites after exposure to high temperature. In this study, the compressive strength of RAC, GMRAC, SFRAC, and SFRHCC was measured at 25 °C as the reference strength. The residual compressive strength was also measured after 3 h of constant-temperature treatment at 80 °C, 150 °C, and 200 °C, followed by cooling to room temperature. The results are shown in Figure 2. The strength retention was calculated as the ratio of the residual compressive strength to the reference compressive strength and is also presented in the figure. At the reference state (25 °C), the incorporation of 3.5 vol% steel fibers increased the apparent density of the composites from approximately 2350 kg/m^3^ (RAC) to 2610 kg/m^3^ (SFRHCC). This substantial density shift physically reflects the embedding of a high-stiffness metallic framework within the matrix, which directly contributes to the notably higher initial compressive strengths of the fiber-reinforced groups (SFRAC and SFRHCC) compared to the baseline RAC. Subsequently, when evaluating the thermal effects, the compressive strength of all mixtures did not decrease after treatment at 80 °C, with strength retention values above 100%. This indicates that heat treatment at relatively low temperatures does not impair the mechanical performance of the matrix. When the treatment temperature increased to 150 °C, the compressive strength of all mixtures began to decrease. RAC showed the largest reduction, reaching 10%, while the reductions in GMRAC and SFRAC were approximately 5%. This indicates that both graphite and steel fibers can partially mitigate the strength degradation caused by thermal damage. The strength reduction of SFRHCC was only about 3%, indicating a strong synergistic strengthening effect of graphite and steel fibers on the mechanical performance of concrete after high-temperature exposure. When the target temperature was further increased to 200 °C, the compressive strength of RAC, GMRAC, and SFRAC decreased sharply, with strength retention values of 75%, 86% and 86%, respectively. In contrast, SFRHCC exhibited the highest strength retention, reaching 92.0%.

### 3.2. Residual Splitting Tensile Strength After Thermal Treatment

**Figure 3:**
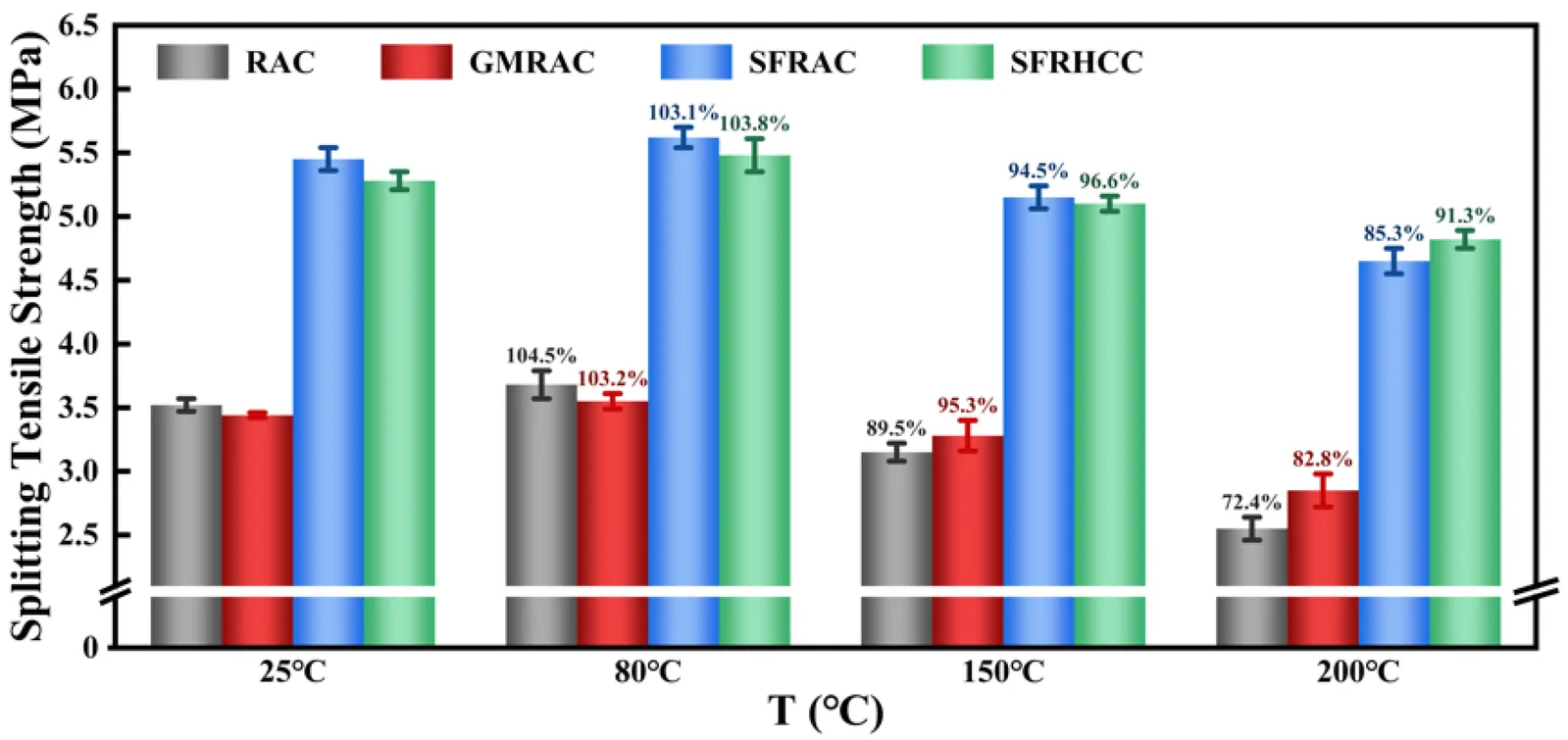
Splitting tensile strength and strength retention rate of various concrete systems at 25, 80, 150, and 200 °C.

Splitting tensile strength is an indirect measure of the tensile failure resistance of materials. It is more sensitive to internal defects, such as microcracks and pore deterioration in the interfacial transition zone (ITZ), and is therefore an important mechanical indicator for evaluating the crack resistance and durability of cement-based composites after exposure to high temperature [26,27]. Figure 3 shows the reference splitting tensile strength of the four concrete mixtures at 25 °C and their residual splitting tensile strengths after 3 h of constant-temperature treatment at 80 °C, 150 °C, and 200 °C, followed by cooling to room temperature. The splitting tensile strength retention was calculated as the ratio of the residual strength to the reference strength.

Similar to the evolution of residual compressive strength, the residual splitting tensile strength of all mixtures exhibited clear stage-dependent characteristics after different thermal treatments. After exposure to 80 °C, the concrete showed an initial thermal strengthening effect, with a slight increase in residual tensile strength. When the treatment temperature increased to 150 °C, the tensile performance began to decrease markedly. This may be attributed to rapid water loss and thermal mismatch stresses at the multiphase interfaces, which promoted the formation of initial microcracks. Under splitting tensile loading, stress concentration at the crack tips induced the propagation of opening cracks [28], resulting in a reduction in splitting tensile strength. After treatment at 200 °C, the residual splitting tensile strength decreased further. RAC exhibited the greatest reduction, with a strength retention of only 72.4%. This indicates that the weak interfaces between the recycled coarse aggregate and the old adhered mortar are more prone to sliding and cracking during high-temperature exposure. The strength retention values of GMRAC and SFRAC were 82.8% and 85.3%, respectively, indicating that both the graphite coating layer and steel fiber reinforcement can partially mitigate interfacial damage. However, statistical analysis revealed no significant difference between these two single-modified groups, as the minor variation falls within experimental uncertainty. Fundamentally, the splitting-tensile and cracking behavior of fiber-reinforced concrete is strongly governed by the fiber content and the associated crack-bridging capacity [29]. After severe thermal damage at 200 °C, these individual modification strategies reach a performance plateau in restraining macro-crack propagation. A statistically distinct enhancement is only realized in the SFRHCC system, which achieved a 91.3% retention. This confirms that a true synergistic effect is established by coupling the stress-accommodating graphite layer with the robust crack-bridging capacity of the steel fibers, collectively preventing catastrophic tensile failure after thermal exposure.

### 3.3. Macroscopic Thermal Expansion Strain During Heating

**Figure 4:**
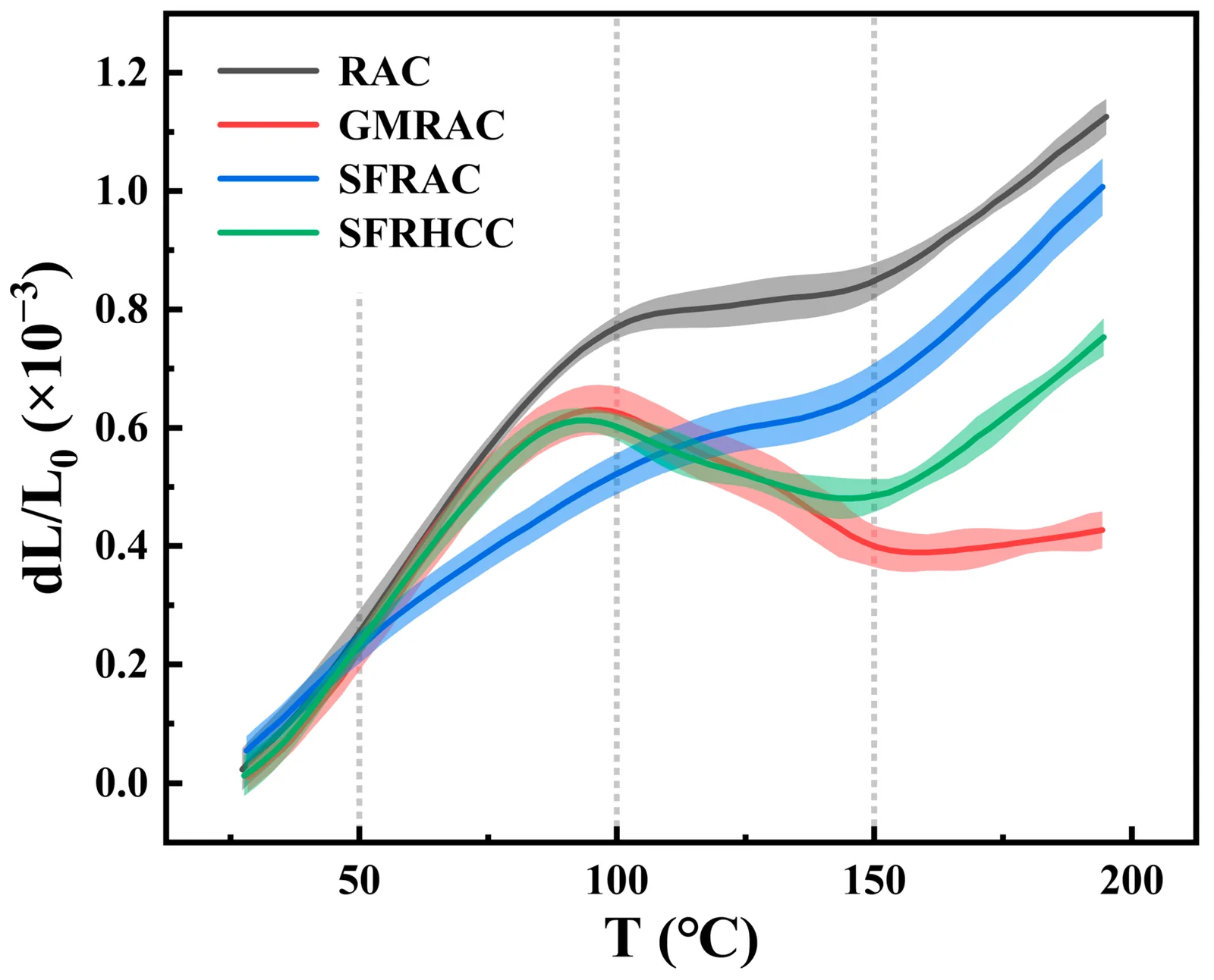
Continuous evolution curves of the thermal expansion strain as a function of temperature for various concrete systems (25–200 °C).

Figure 4 shows the macroscopic thermal expansion strain curves of RAC, GMRAC, SFRAC, and SFRHCC during heating from 25 to 200 °C. Based on the thermo-mechanical responses of the multiphase components across different temperature ranges, the deformation process shows a continuous transition from liquid–solid coupled deformation to thermal expansion of the solid phase. According to the characteristics of the deformation evolution, this process is divided into three stages: the low-temperature stage (25–100 °C), the intermediate-temperature stage (100–150 °C), and the high-temperature stage (150–200 °C).

In the low-temperature stage, the thermal deformation of concrete is mainly governed by the thermal expansion of free water and vapor pressure within the pores. In the initial range of 25–50 °C, SFRAC exhibits the highest macroscopic deformation rate. This is mainly attributed to the incorporation of highly conductive steel fibers, which increases the heat-transfer rate within the specimen. In addition, the intrinsic linear thermal expansion coefficient of steel fibers (11–13 × 10^−6^/°C) is slightly higher than that of ordinary concrete (10–12 × 10^−6^/°C), resulting in a faster volumetric expansion response upon heating [17]. In contrast, the initial deformation of the graphite-containing specimens (GMRAC and SFRHCC) is relatively low. This is because the layered structure of graphite provides local space for deformation at the microscale [30], effectively accommodating and absorbing the initial local expansion stresses caused by thermal mismatch between the different components. As the temperature further increases to 50–100 °C, heating of the free water in the pores generates saturated vapor pressure, causing a rapid increase in the thermal expansion strain of RAC, with the curve slope reaching a peak in this stage. In SFRAC, however, the high-stiffness steel fiber network provides an effective bridging effect and mechanically constrains the thermal expansion of the matrix, resulting in a clear reduction in the expansion rate [31,32]. Overall, the thermal deformation of the composites in this stage is mainly governed by the competition between heat-transfer-induced expansion and mechanical restraint from the steel fibers.

In the intermediate-temperature stage, significant physical dehydration and water evaporation occur within the composites, leading to pronounced drying shrinkage of the matrix. The macroscopic thermal deformation therefore results from the dynamic competition between solid phase thermal expansion and volume contraction caused by water loss. During this stage, the thermal expansion strain of SFRAC continues to increase, indicating that the high-stiffness steel fiber network forms a strong supporting framework within the matrix. Its mechanical restraint is sufficient to resist the capillary shrinkage stress caused by water loss, indicating that the fiber restraint is greater than the capillary shrinkage force. RAC shows an obvious deformation plateau, where thermal expansion of the solid phase and drying shrinkage caused by water loss from the gel pores are approximately balanced, resulting in a relatively stable net deformation. GMRAC and SFRHCC, in contrast, show pronounced volumetric contraction in this temperature range. This may be attributed to the layered structure of graphite, which provides low resistance pathways for the outward migration of water vapor and accelerates water loss. As a result, the capillary tension and shrinkage caused by water loss become dominant.

In the high-temperature stage, free water and most physically adsorbed water have essentially evaporated, and the deformation behavior is dominated by the thermo-mechanical response of the solid phases under dry conditions. SFRHCC, SFRAC, and RAC recover a stable linear expansion behavior, with their macroscopic expansion rates mainly determined by the intrinsic thermal expansion coefficients of the hardened cement paste, aggregates, and steel fibers. In contrast, the expansion curve of GMRAC becomes nearly horizontal, indicating an extremely low macroscopic thermal expansion coefficient. This intrinsic thermal behavior suggests that in the absence of a three-dimensional rigid support provided by the steel fiber network, the low intrinsic thermal expansion of graphite (−1.8 × 10^−6^/°C [33]) and the abundant microscopic spaces between its layers allow graphite to continuously accommodate the thermal deformation of the surrounding matrix. This gives GMRAC excellent volumetric stability at high temperatures.

In summary, the macroscopic thermal expansion behavior of the different concrete systems during heating from 25 to 200 °C exhibits clear stage-dependent characteristics. The evolution of strain is governed by the dynamic competition and interaction among intrinsic thermal expansion of the solid phases, pore vapor pressure, and drying shrinkage caused by water loss. GMRAC takes advantage of the porous layered structure of graphite to continuously accommodate thermal mismatch stresses from the matrix during the initial heating and high temperature stages. In the intermediate-temperature stage, it also provides low-resistance pathways for water vapor transport, accelerating water evaporation and making capillary shrinkage the dominant deformation mechanism. SFRAC relies on its high-stiffness fiber network to provide effective mechanical restraint and bridging at low and intermediate temperatures. However, it undergoes continuous linear expansion at high temperatures due to the relatively high thermal expansion of its constituent phases. In contrast, SFRHCC achieves effective coupling of the thermal deformation of the different phases over the entire temperature range. Through local restraint and deformation coordination among the phases, SFRHCC effectively suppresses large fluctuations in macroscopic volume at different temperatures and exhibits excellent high-temperature volumetric stability. This stability is also an important factor contributing to its superior mechanical performance after high-temperature treatment.

### 3.4. Variation of Thermal Conductivity After Thermal Treatment

**Figure 5:**
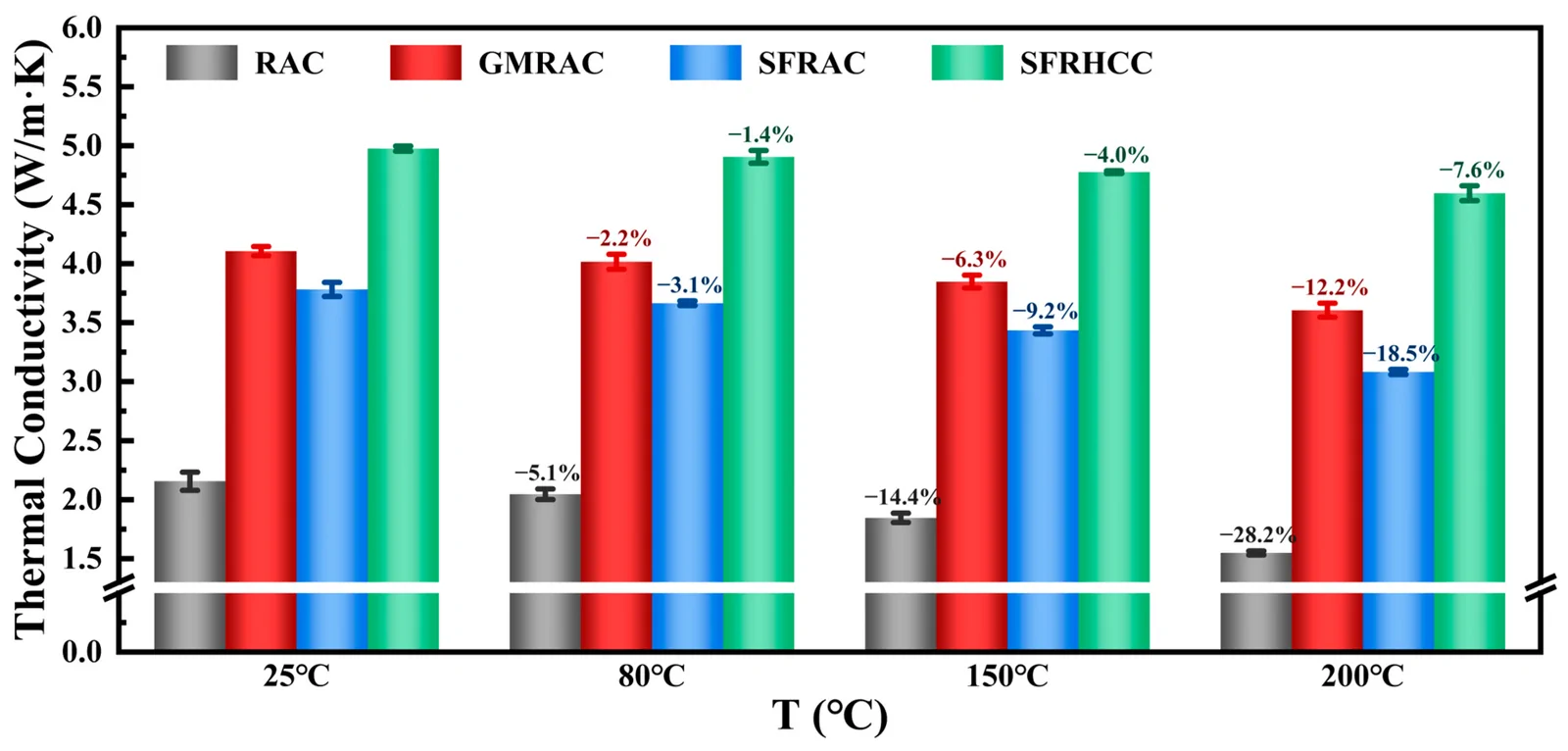
Evolution and high-temperature reduction amplitude of the thermal conductivity for various concrete systems from 25 to 200 °C.

Figure 5 shows the thermal conductivity of RAC, GMRAC, SFRAC, and SFRHCC after 3 h of heat treatment at different target temperatures, followed by cooling to room temperature. The corresponding reductions relative to the initial thermal conductivity at 25 °C are also presented. As shown in the figure, all concrete systems experienced thermal damage after heat treatment, resulting in a clear reduction in thermal conductivity. The higher the treatment temperature, the greater the reduction. This is mainly because heating causes irreversible thermal damage, including the formation of microcracks and deterioration of the pore structure. These defects act as strong phonon-scattering centers, shortening the phonon mean free path. The increased phonon scattering at the multiphase interfaces further reduces the residual thermal conductivity of all systems [34,35,36].

Both graphite and steel fibers substantially improve the stability of thermal conductivity after high-temperature exposure and effectively slow its degradation. The reductions in thermal conductivity of GMRAC after treatment at 80, 150, and 200 °C were 2.2%, 6.3% and 12.2%, respectively, which were 0.9%, 2.9% and 6.3% lower than those of SFRAC, respectively. This indicates that graphite provides better retention of thermal conductivity after high-temperature exposure than steel fibers. When graphite and steel fibers were used together, the reduction in the thermal conductivity of SFRHCC was further suppressed. After treatment at 200 °C, its residual thermal conductivity decreased by only 7.6% relative to the initial value, showing the best thermal conductivity retention among the four systems. These results demonstrate that both the graphite coating layer and steel fibers improve the intrinsic thermal conductivity of RAC, while their combination further enhances this effect by forming a continuous multiphase heat-transfer network. The composite conductive network not only improves heat transfer but also exhibits strong resistance to thermal degradation, thereby maintaining continuous and stable heat transfer pathways after high-temperature exposure.

### 3.5. Spatial Distribution of the Synergistic Heat-Transfer Network

**Figure 6:**
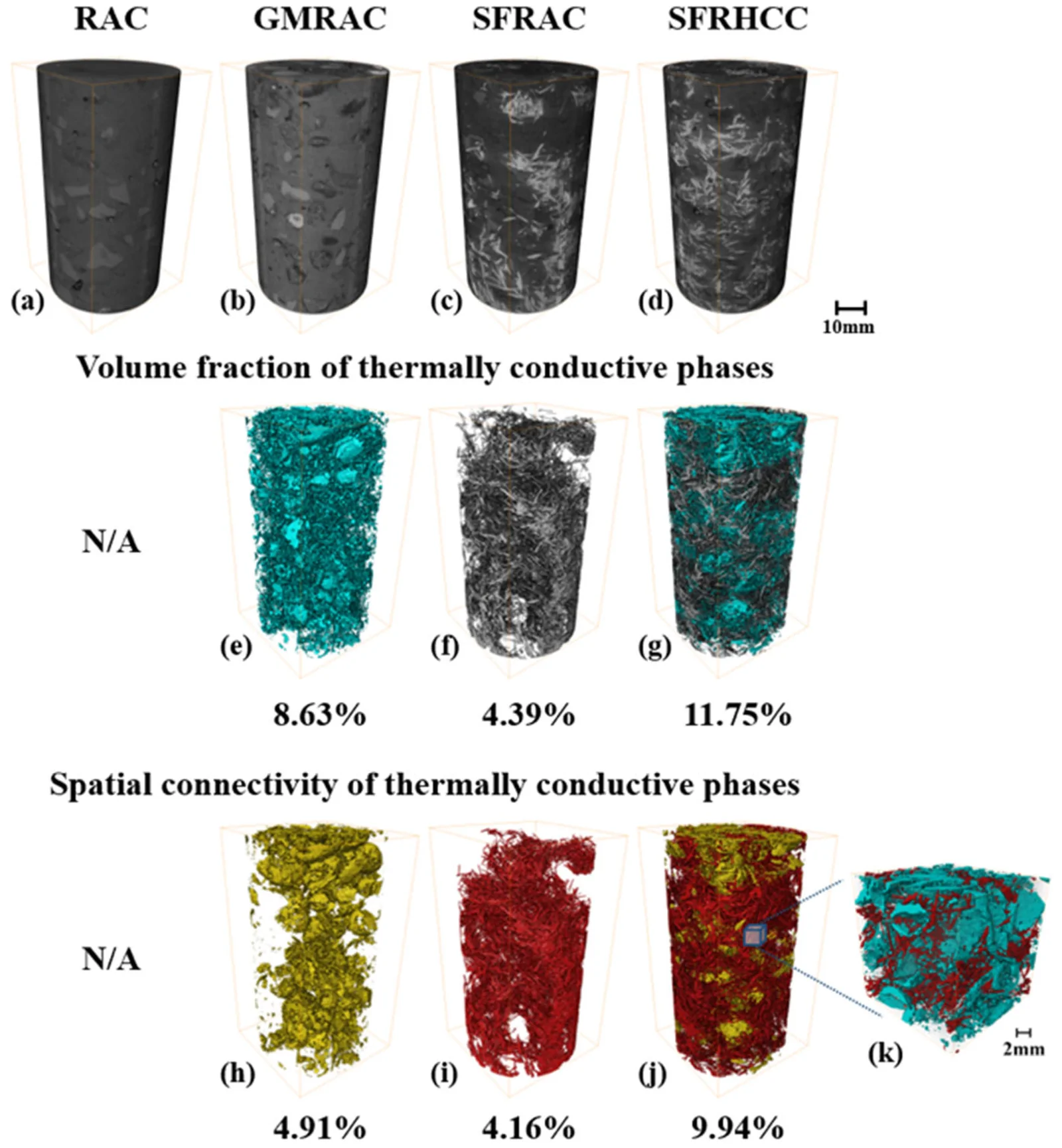
X-CT 3D spatial reconstruction and connected network extraction of internal thermally conductive phases of different concrete systems. (**a**) RAC group: overall 3D reconstruction of the specimen; (**b**,**e**,**h**) GMRAC group: overall 3D reconstruction of the specimen, spatial distribution of graphite coating layer, and connected graphite coating layer network, respectively; (**c**,**f**,**i**) SFRAC group: overall 3D reconstruction of the specimen, spatial distribution of steel fiber, and connected steel fiber network, respectively; (**d**,**g**,**j**) SFRHCC group: overall 3D reconstruction of the specimen, spatial distribution of mixed high thermally conductive phase (graphite coating layer + steel fiber), and connected mixed thermally conductive network, respectively; (**k**) local magnified micro-morphology of the interface area between the steel fiber and graphite coating layer. “N/A” indicates the RAC group contains no thermally conductive phases.

Figure 6 shows the three-dimensional X-CT reconstructed images of RAC, GMRAC, SFRAC, and SFRHCC. The distinct density differences among the constituent phases allow each phase to be accurately extracted using grayscale threshold segmentation. As shown in Figure 6b,e,h, the highly conductive graphite coating layer in GMRAC is distributed as a shell around the coarse aggregates. However, due to the spatial distance between aggregates and the physical separation imposed by the cement matrix, the three-dimensional connectivity of the graphite coating layer alone is only 4.91%. Thus, although the graphite coating layer is uniformly distributed throughout the concrete, it lacks long-range conductive pathways and cannot efficiently transfer heat across the specimen. In SFRAC (Figure 6c,f,i), the connectivity of the steel fibers is slightly lower, at only 4.16%. This indicates that the one-dimensional fibers are also unable to form an efficient continuous network within the matrix, consistent with the residual thermal conductivity results in Section 3.4. In contrast, SFRHCC shows a markedly different spatial arrangement of the constituent phases (Figure 6d,g,j). A three-dimensional network formed by the overlap and connection of linear and planar phases can be clearly observed, with the overall connectivity increasing to 9.94%. This value exceeds the combined connectivity of the individual phases (9.07%), indicating a clear spatial synergy between the two phases. Fundamentally, the spatial orientation of steel fibers strictly governs the directional efficiency of heat and stress transfer. According to classical micromechanical models [37], the theoretical efficiency factor of three-dimensionally random short fibers is exactly one-third of that for fully aligned fibers, highlighting the massive anisotropic potential of fiber-reinforced systems. However, as visually confirmed by the 3D X-CT reconstruction in this study, the relatively short steel fibers (5 mm) were sterically constrained by the large coarse aggregates (up to 16 mm) and forced into a spatially random 3D overlapping network during vibration casting. Consequently, the severe macroscopic anisotropy typically induced by preferential fiber alignment is volumetrically averaged out, yielding a macroscopically isotropic system. Thus, the thermal conductivity and mechanical strengths evaluated in this study reliably represent the effective bulk properties of the composite.

The enlarged image of SFRHCC in Figure 6k further reveals the geometric origin of this synergy. In the local contact regions, the ends or middle sections of the steel fibers are in direct contact with the graphite coating layer. In this configuration, the high aspect ratio steel fibers act as physical bridges across the interfaces, connecting the otherwise isolated graphite coating layers over longer distances. Through the spatial interconnection of the one-dimensional steel fibers and two-dimensional graphite layers, a hybrid heat-transfer skeleton is formed throughout the specimen. This interconnected structure is a major factor contributing to the superior residual thermal conductivity of SFRHCC.

**Figure 7:**
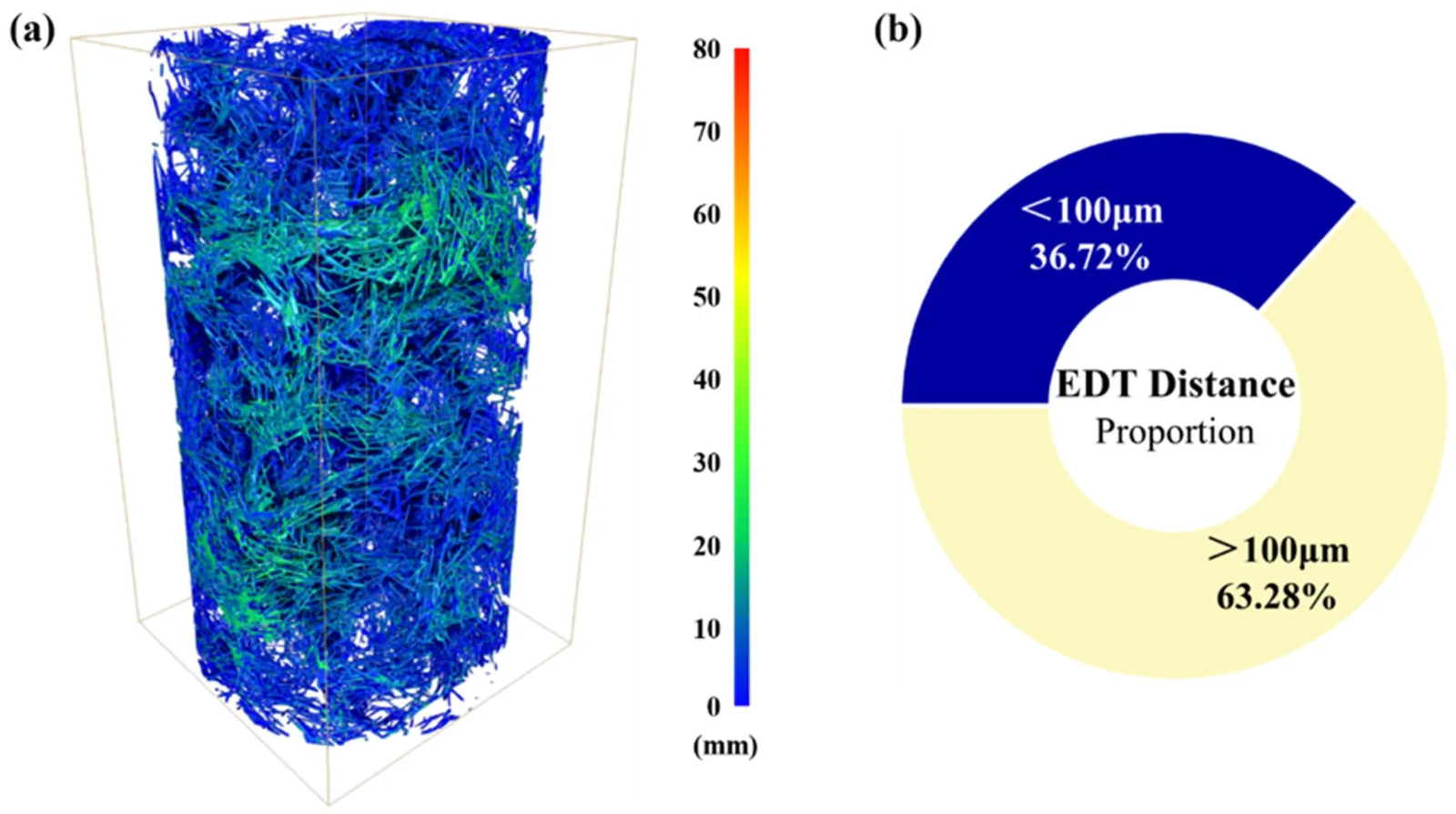
Euclidean distance transform analysis of the spatial distances between the steel fibers and the graphite coating layer: (**a**) 3D color map of the minimum distance from the steel fibers to the graphite coating layer; (**b**) statistical distribution of the proportions of different spatial distances between the steel fibers and the graphite coating layer.

To further quantify the near contact characteristics between steel fibers and the graphite coating layer in SFRHCC, the EDT was applied. The minimum spatial distance from each point on the steel fiber surface to the nearest graphite coating layer was calculated, yielding a three-dimensional visualization and quantitative distribution of the spatial distances, as shown in Figure 7.

In Figure 7a, the color variation of the steel fibers represents their distance from the graphite coating layer. Considering the interfacial phonon scattering and the effective attenuation length of heat transfer, the contribution of phases separated by large distances to the macroscopic thermal conductivity can be considered negligible. Therefore, based on the physical principles of microscale heat transfer, a critical distance of 100 μm was selected to define effective near contact between the linear and planar phases. This threshold precisely corresponds to the typical maximum thickness of the interfacial transition zone (ITZ) in concrete. Given the relatively low intrinsic thermal conductivity of the bulk cement paste (approximately 1.1 W/m·K), physical distances exceeding this microstructural scale introduce severe localized thermal resistance (Kapitza resistance) and massive phonon scattering. Conversely, when the distance is within 100 μm, the highly conductive phases are sufficiently close to bridge the thermal barrier of the intervening paste, significantly reducing this resistance and allowing efficient local heat-transfer pathways to form.

As shown in Figure 7b, 36.72% of the steel fiber volume in the reconstructed three-dimensional region is located within 100 μm of the graphite coating layer. This indicates that more than one-third of the steel fibers are in very close proximity to, or directly connected with, the graphite coating layer. The high proportion of near neighbor contacts confirms the formation of a linear–planar interconnected network. A substantial fraction of the steel fibers bridges the spatial separation imposed by the cement matrix and forms dense local heat-transfer pathways with the graphite phase. This network is an important reason for the notable improvement in the macroscopic thermal conductivity of the composite system. In summary, the X-CT and EDT analyses provide direct structural evidence of a highly interconnected multi-dimensional network (e.g., the 9.94% effective near-contact volume), while the macroscopic tests quantitatively confirm the enhanced thermo-mechanical stability of the SFRHCC system. This verified physical proximity of the conductive phases is inferred to facilitate continuous phonon transport for heat dissipation and enable localized stress redistribution. Consequently, this microstructural synergy is suggested to be the primary mechanism enhancing the thermo-mechanical performance of the composites.

### 3.6. Interfacial Contact Morphology of the Synergistic Heat-Transfer Network

The actual heat-transfer efficiency of the composite system depends not only on the formation of a macroscopically connected network, but also on the quality of microscale contact at multiphase interfaces. Poor interfacial contact introduces high thermal resistance and limits the enhancement of thermal conductivity. In this system, although the graphite coating layer is closely integrated with the concrete matrix, the contact quality between the steel fiber network and the matrix is also critical to heat transfer. To clarify the mechanism underlying the synergistic enhancement of thermal conductivity by graphite and steel fibers, scanning electron microscopy (SEM) and energy dispersive spectroscopy (EDS) were used to characterize the interfacial contact morphology of the multiphase components in the steel fiber-containing systems.

**Figure 8:**
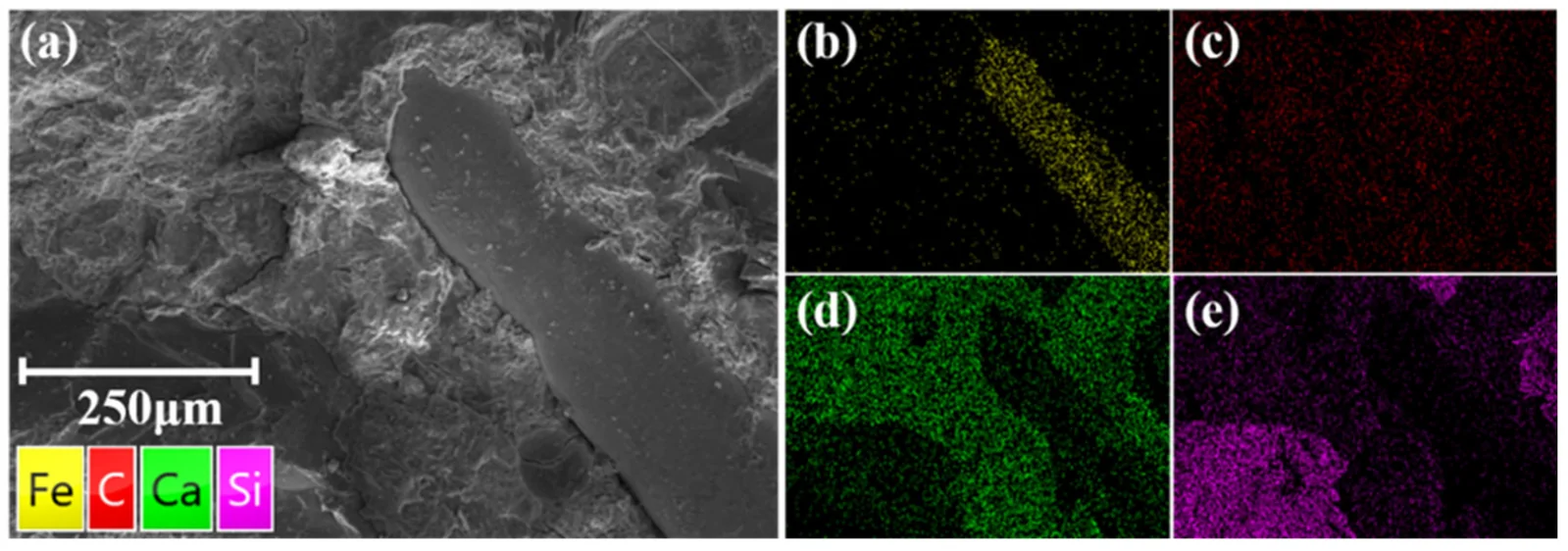
Micro-morphology and elemental mapping of the interface between steel fibers and recycled coarse aggregates in the SFRAC system: (**a**) SEM image of the interface; (**b**) EDS mapping of Fe element; (**c**) EDS mapping of C element; (**d**) EDS mapping of Ca element; (**e**) EDS mapping of Si element.

Figure 8 shows the SEM-EDS results of SFRAC. Although the steel fibers form a heat-transfer skeleton within the cement matrix, they are not in close contact with the hardened cement matrix. In addition, the cement matrix has a relatively low thermal conductivity of approximately 1.1 W/(m·K) [38], which hinders heat transfer when heat reaches the boundary of the recycled coarse aggregate. These high resistance interfaces further increase phonon scattering and reduce heat exchange between the steel fibers and coarse aggregates. As a result, efficient heat-transfer pathways across different length scales are difficult to establish, limiting improvement in the macroscopic thermal conductivity of the system [39].

**Figure 9:**
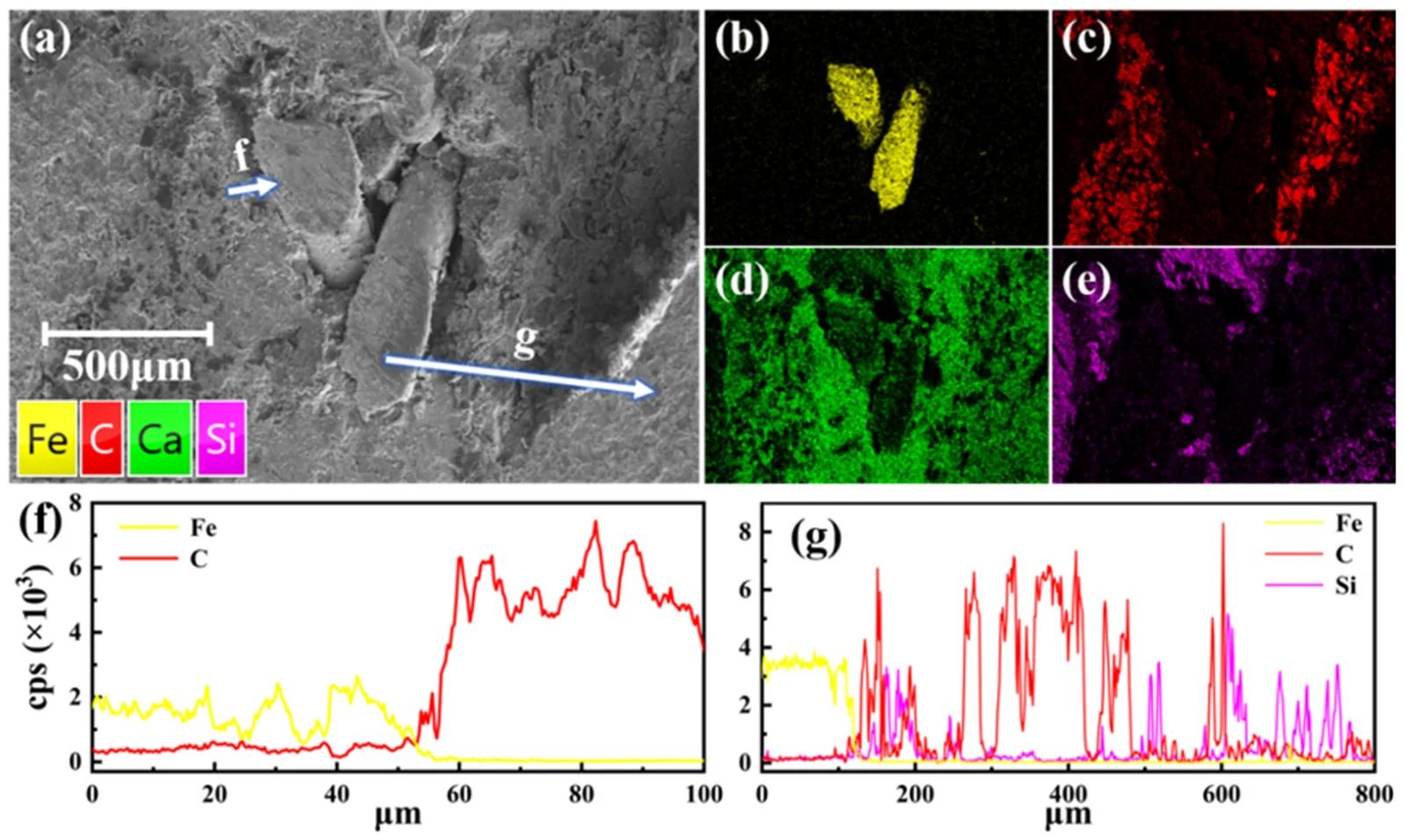
Micro-morphology and EDS elemental analysis of the “ternary contact zone” in the SFRHCC system: (**a**) microstructure of the “steel fiber–graphite coating layer–coarse aggregate” interface, where the white arrows labeled ‘f’ and ‘g’ indicate the specific paths for the EDS line scans; (**b**–**e**) corresponding elemental mapping images of Fe, C, Ca, and Si in the micro-region; (**f**,**g**) corresponding elemental distribution gradient curves across the interface along paths f and g, respectively.

Figure 9 shows the SEM-EDS results of SFRHCC. As shown in the micrograph in Figure 9a, the graphite coating layer is closely bonded to the cement matrix, with no obvious gaps observed at the interface. Moreover, the contact between the steel fibers and the soft graphite coating layer is better than that between the steel fibers and the hardened cement matrix (see Figure 8). The steel fibers are embedded in the matrix and closely contact the graphite coating layer on the surface of the recycled coarse aggregates, providing a favorable basis for heat transfer in SFRHCC. To verify the continuity of the phase distribution in this region, EDS elemental mapping was performed. The elemental maps in Figure 9b–e show the distribution of the main elements across the interface. The carbon element, representing the graphite coating layer, forms a continuous and well-defined enriched region between the steel fiber and the coarse aggregate substrate. An EDS line scan was then performed along the arrow shown in Figure 9a. As shown in Figure 9f,g, the signal intensities of the characteristic elements show a gradual transition across the interface. The iron signal from the steel fiber decreases sharply at the interface, while the carbon signal from the graphite coating layer decreases gradually and continuously. This stepwise variation in elemental concentration confirms, at the microscale, the formation of a close contact interface between the highly conductive phases.

### 3.7. Dissipation of Interfacial Thermal Mismatch Stress

The macroscopic thermal expansion results in Section 3.3 show that SFRHCC exhibits excellent volumetric stability and a low thermal expansion coefficient after high-temperature exposure, whereas the single modified systems show more pronounced thermal damage. At high temperatures, the differences in thermal expansion coefficients among the steel fibers, cement matrix, and recycled coarse aggregates generate significant thermal mismatch stresses, with the steel fibers being a major source of this mismatch. Therefore, this section focuses on the two steel fiber-containing systems, SFRAC and SFRHCC, to clarify the evolution and dissipation of thermal mismatch stress. GMRAC was not considered here because the low thermal expansion and deformation accommodating ability of graphite lead to a different thermal response from that of steel fibers. SEM-EDS was used to characterize the microcracks and interfacial morphology of SFRAC and SFRHCC after heat treatment.

**Figure 10:**
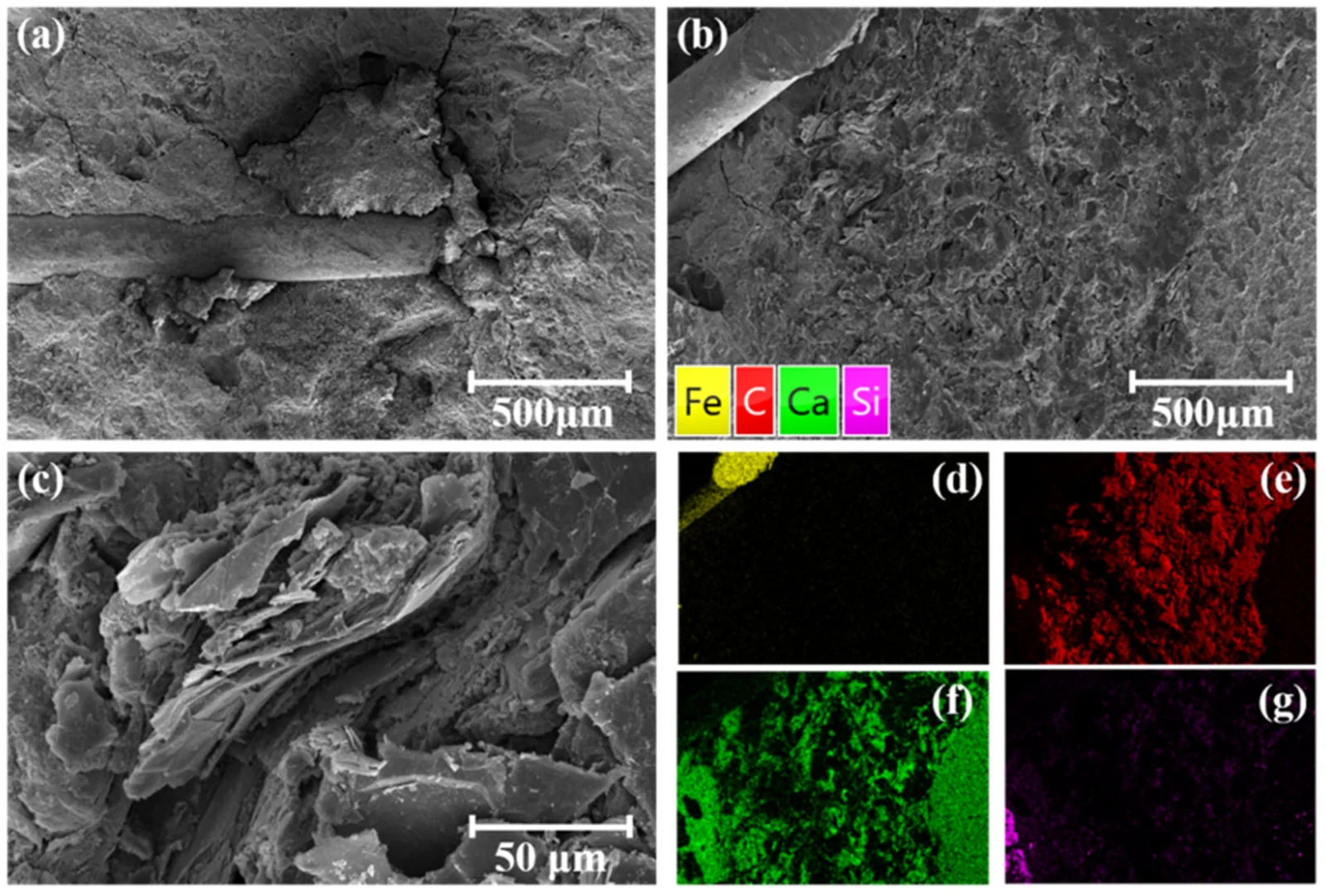
Internal thermal mismatch damage characteristics and stress dissipation mechanism of the graphite layer in the composites after exposure to 200 °C: (**a**) SFRAC system: morphology of matrix microcrack initiation and propagation; (**b**) SFRHCC system: morphology of interfacial overlapping and micro-damage localization; (**c**) microscopic characteristics of interlayer sliding and compressive deformation of the graphite coating layer in (**b**); (**d**–**g**) corresponding EDS elemental mapping images of the micro-region in (**b**) (distributions of Fe, C, Ca, and Si).

Figure 10a shows the microstructure of SFRAC after treatment at 200 °C. Because both the recycled coarse aggregates and steel fibers undergo substantial thermal expansion, the resulting thermal mismatch generates concentrated local stresses in the brittle cement matrix. These stresses cause damage in the matrix and weak interfacial regions, leading to microcrack formation and further crack propagation under thermal stress. This is one of the reasons for the reduction in compressive strength after high-temperature treatment. In contrast, the introduction of the highly conductive graphite coating layer in SFRHCC changes the stress transfer path and damage evolution at the interface. Figure 10b shows the microstructure of the steel fiber–graphite coating–coarse aggregate region after high-temperature treatment. Combined with the corresponding EDS elemental maps in Figure 10d–g, the cement matrix remains largely intact, without the extensive through cracks observed in SFRAC. A continuous and well-defined carbon-rich layer, corresponding to the graphite coating layer, separates the iron-rich region of the steel fiber from the calcium and silicon-rich regions of the matrix and aggregate. Even after the shear deformation induced by heating, the carbon-rich network remains continuous, without obvious chemical separation or large-scale debonding.

Figure 10c provides an enlarged view of the graphite coating layer in Figure 10b. The graphite coating layer confined between the rigid aggregate and cement matrix undergoes clear nonlinear compression and significant interlayer sliding. This behavior is mainly attributed to the unique hexagonal layered structure of graphite and its extremely low interlayer friction coefficient [40,41,42]. During heating, when the recycled coarse aggregate undergoes volumetric expansion, the resulting rigid displacement is not directly transferred to the surrounding cement matrix. Instead, it is accommodated by the collapse and sliding of the graphite layers. Thus, the graphite coating layer provides an effective stress dissipation zone for the thermal mismatch between the steel fibers and cement matrix. It absorbs and buffers most of the thermal mismatch stress, thereby improving the mechanical stability of the matrix after high-temperature exposure.

### 3.8. Mechanical Restraint of Steel Fibers and Failure Mode Transition

**Figure 11:**
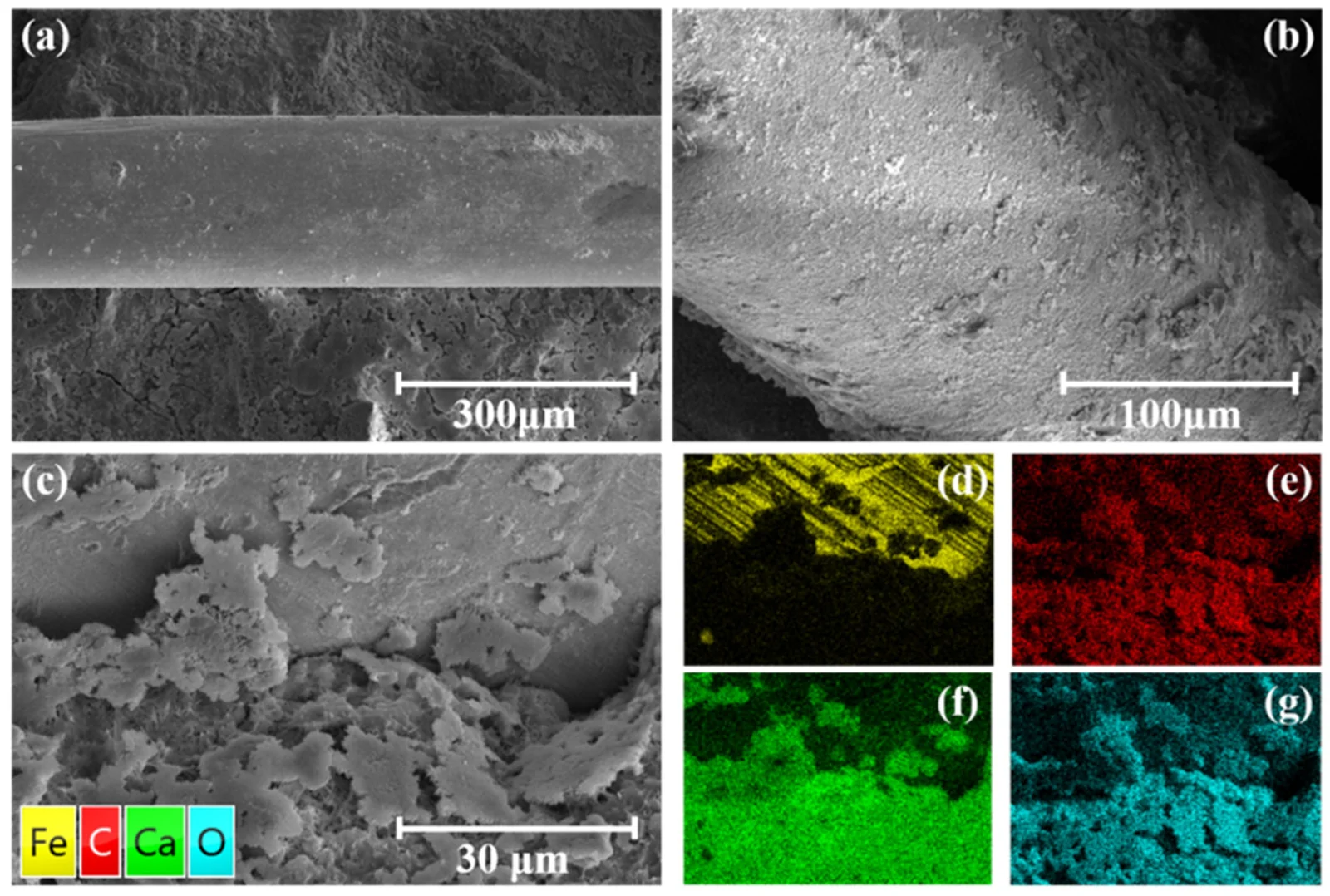
Surface morphology and elemental distribution of pulled-out fibers after 200 °C treatment, cooling, and failure: (**a**) morphology of simple debonding on the fiber surface in the SFRAC system; (**b**) morphology of the roughened fiber surface and attached hydration products in the SFRHCC system; (**c**) locally magnified micro-morphology of the attached products on the fiber surface in (**b**); (**d**–**g**) corresponding EDS elemental mapping images of the micro-region in (**c**) (distributions of Fe, C, Ca, and O).

Figure 11a shows the surface morphology of the steel fiber pulled out from the SFRAC matrix after fracture. In the conventional system, fiber-matrix debonding mainly occurs through simple interfacial separation. The steel fiber has a relatively smooth surface after pull-out, with little matrix residue or hydration products attached. This indicates that high-temperature exposure causes severe microstructural loosening and thermal damage at the fiber–matrix interface, weakening both mechanical interlocking and chemical bonding. Under the applied stress field, cracks can easily propagate along the weak sliding interface. The fiber mainly dissipates energy through limited interfacial friction, resulting in substantial degradation of the residual mechanical properties [43].

In contrast, the pulled-out fibers in SFRHCC exhibit distinctly different failure characteristics. As shown in Figure 11b, the steel fiber surface does not show large smooth debonded regions. Instead, it exhibits high surface roughness with a large amount of dense hydration products attached. This indicates that the stress-buffering effect of the graphite coating layer helps preserve the density and structural continuity of the cement matrix after high-temperature exposure, allowing it to maintain effective lateral restraint on the steel fiber. Fundamentally, the macroscopic bridging efficiency and microscopic pull-out behavior of cementitious composites are significantly governed by the fiber geometry and aspect ratio [44]. Short fibers with a relatively low aspect ratio (L/D = 25), while beneficial for ensuring uniform dispersion at a high dosage of 3.5 vol%, inherently possess a shorter embedment length and are typically more susceptible to simple pull-out under stress. However, in the SFRHCC system, the preserved intact matrix effectively compensates for this geometric limitation by providing strong lateral gripping. Consequently, the interfacial shear failure mode changes from simple weak interface debonding to high-energy cohesive failure extending into the cement matrix.

Figure 11c shows an enlarged view of the steel fiber in Figure 11b. The attached hydration products form an interlocking structure on the fiber surface. EDS elemental mapping was further performed in this region, as shown in Figure 11d–f. The characteristic elements of the hydration products show clearly defined enriched regions, indicating dense contact between the hydration products and the steel fiber. These results further confirm that severe mechanical interlocking and matrix tearing occur on both sides of the sliding interface during fiber pull-out. This mechanism contributes to the superior splitting tensile performance of SFRHCC after high-temperature treatment, as discussed in Section 3.2.

### 3.9. Mechanisms of Thermo-Mechanical Enhancement

**Figure 12:**
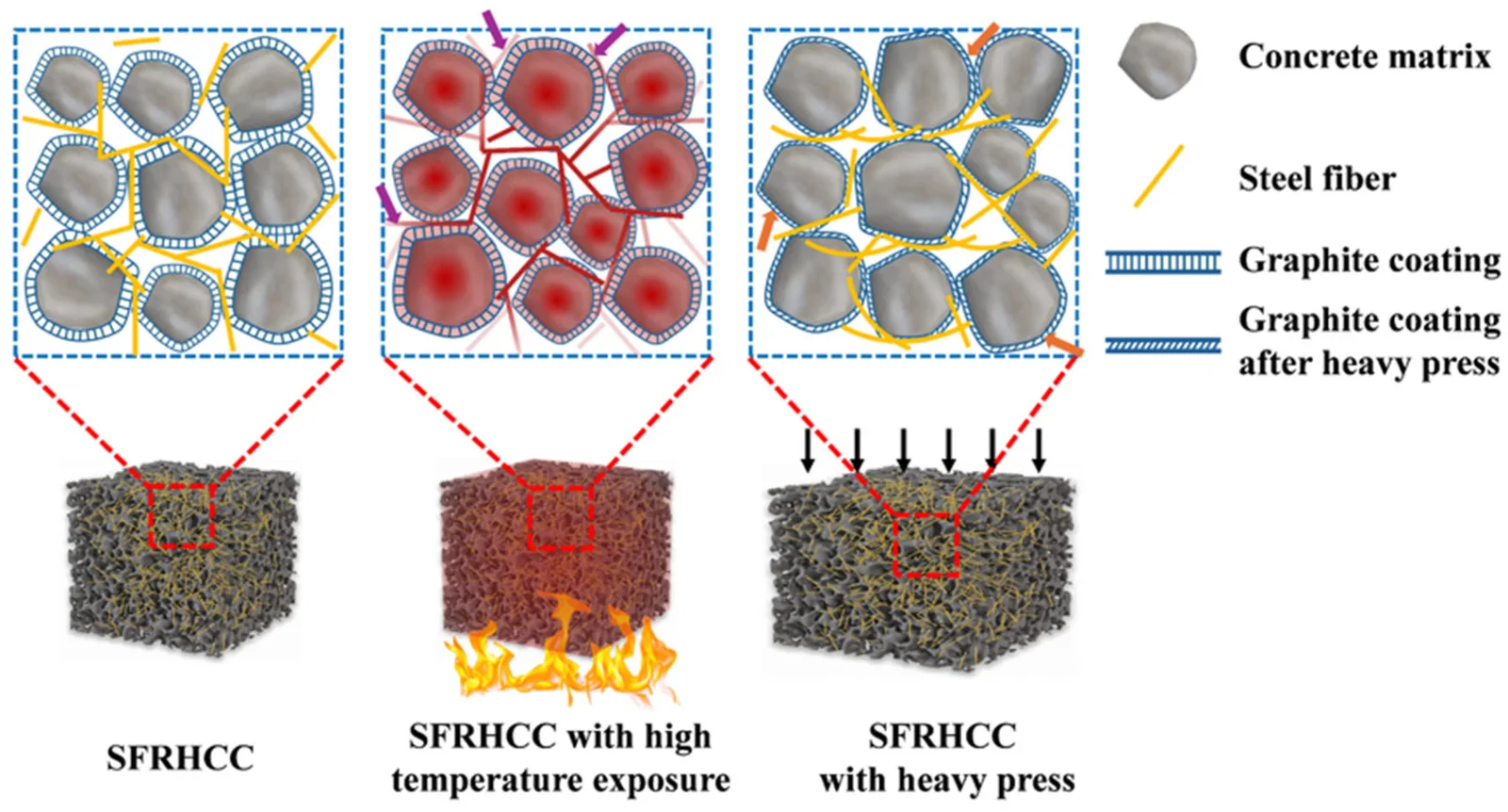
Schematic illustration of the heat-transfer and stress-dissipation mechanisms in SFRHCC during high-temperature exposure and mechanical loading.

The graphite-steel fiber composite recycled concrete developed in this study exhibited excellent mechanical property retention after high-temperature exposure. After treatment at 200 °C for 3 h, the residual compressive and splitting tensile strength retention values both exceeded 90%. The synergistic enhancement mechanisms after high-temperature exposure were characterized using X-CT three-dimensional reconstruction, EDT analysis, and SEM-EDS, as summarized in Figure 12.

As shown in Figure 12, SFRHCC contains a continuous steel fiber-graphite structure, in which the two phases are interconnected to form a three-dimensional network. During high-temperature exposure, the rigid steel fibers and cement matrix develop substantial thermal mismatch stresses. Once these stresses exceed the tensile capacity of the matrix, microcracks can readily initiate and propagate at the ITZs between the matrix and aggregates, leading to mechanical degradation. As discussed in Section 3.4, Section 3.5 and Section 3.6, the highly conductive network formed by graphite and steel fibers rapidly transfers heat away from the cement matrix, as indicated by the purple arrows in Figure 12, thereby reducing thermal mismatch stress. In addition, the layered porous structure and low thermal expansion of the graphite coating layer allow it to accommodate and buffer local thermal mismatch deformation between the coarse aggregates and cement matrix. This suppresses crack initiation and enables SFRHCC to maintain excellent mechanical stability and structural integrity under high-temperature exposure.

As indicated by the orange arrows in Figure 12, the graphite coating layer also dissipates stress through interlayer sliding, interrupting the propagation of stress concentrations and thermally induced cracks into the brittle cement matrix. This mechanism converts potentially harmful macroscopic thermal mismatch displacement into microscale interlayer sliding, thereby dissipating stress and enabling a more gradual transfer of deformation within the composite, as discussed in Section 3.7. Furthermore, the graphite coating layer promotes denser contact between the steel fibers and cement matrix. Under tensile loading, the failure mode therefore changes from simple fiber pull-out to matrix tearing, which substantially increases energy dissipation and toughness. This enhanced interfacial mechanical behavior is another important reason for the excellent retention of mechanical properties after high-temperature exposure.

## 4. Conclusions

In this study, the thermo-mechanical properties and synergistic enhancement mechanisms of concrete incorporating graphite-coated recycled aggregates and steel fibers (SFRHCC) were systematically investigated after thermal treatment up to 200 °C. Based on the experimental results and microstructural analyses, the main conclusions are drawn as follows:

1. The synergistic modification significantly improves the macroscopic thermal and mechanical stability of the concrete. Following thermal treatment at 200 °C, SFRHCC restricted its thermal conductivity loss to only 7.6%. Furthermore, it exhibited the highest mechanical retention among all tested groups, retaining 92.0% of its reference compressive strength and 91.3% of its splitting tensile strength, while effectively restraining severe volumetric fluctuations during the heating process.
2. The outstanding macroscopic performance is fundamentally underpinned by the formation of a highly connected multi-dimensional thermal network. X-CT and EDT analyses confirmed a high-density linear-planar overlap, with 36.72% of the steel fibers located within 100 μm of the graphite coating layer. This spatial proximity elevated the overall spatial connectivity to 9.94%, effectively overcoming the isolation defects of single-phase modifications.
3. A rigid–flexible coupled stress dissipation mechanism is primarily responsible for the superior thermo-mechanical stability after thermal treatment. The soft graphite coating layer accommodates thermal mismatch deformations between the rigid aggregates and the cement matrix via microscopic interlayer sliding. This localized stress dissipation preserves the structural integrity of the surrounding cement matrix. Consequently, the intact matrix supplies robust lateral mechanical constraint to the steel fibers, effectively transforming the interfacial failure mode from simple weak-interface debonding to high-energy cohesive matrix tearing.

These findings provide new insights and a fundamental materials design strategy for developing high-performance thermally conductive concrete capable of maintaining structural integrity and efficient heat transfer in moderate thermal environments. However, the current investigation is primarily limited to steady-state thermal treatments up to 200 °C. Future research should evaluate the long-term durability of the composite under repeated thermal cycling and investigate its degradation mechanisms under extreme high-temperature conditions to further broaden its practical application scope.

## Figures and Tables

**Table 1:** Mixture design of concrete specimens (kg/m^3^).

Sample	Cement	Water	Fine Aggregate	Recycled Coarse Aggregate	Heat-Conductive Coarse Aggregate	Steel Fiber (vol%)
RAC	500	300	560	1040	/	/
SFRAC	500	300	560	1040	/	3.5
GMRAC	500	300	560	/	1040	/
SFRHCC	500	300	560	/	1040	3.5

## Data Availability

The original contributions presented in this study are included in the article. Further inquiries can be directed to the corresponding authors.
